# Enhancing the Catalytic Activity of Palladium Nanoparticles via Sandwich-Like Confinement by Thin Titanate Nanosheets

**DOI:** 10.1021/acscatal.1c00031

**Published:** 2021-02-15

**Authors:** Kevin Ament, Daniel R. Wagner, Thomas Götsch, Takayuki Kikuchi, Jutta Kröhnert, Annette Trunschke, Thomas Lunkenbein, Takayoshi Sasaki, Josef Breu

**Affiliations:** † Bavarian Polymer Institute and Department of Chemistry, University of Bayreuth, 95447 Bayreuth, Germany; ‡ Department of Inorganic Chemistry, Fritz-Haber-Institut der Max-Planck-Gesellschaft, Faradayweg 4-6, 14195 Berlin, Germany; § International Centre for Materials Nanoarchitectonics (WPI-MANA), National Institute for Materials Science (NIMS), 1-1 Namiki, Tsukuba, Ibaraki 305-0044, Japan

**Keywords:** layered titanate, palladium nanoparticles, CO oxidation, support−metal interaction, heterogeneous catalysis

## Abstract

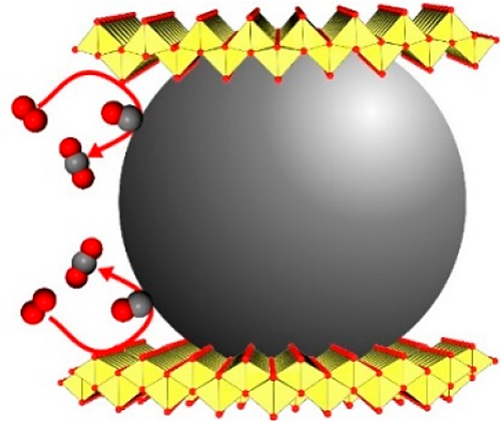

As atomically thin oxide layers deposited on flat (noble) metal surfaces have been proven to have a significant influence on the electronic structure and thus the catalytic activity of the metal, we sought to mimic this architecture at the bulk scale. This could be achieved by intercalating small positively charged Pd nanoparticles of size 3.8 nm into a nematic liquid crystalline phase of lepidocrocite-type layered titanate. Upon intercalation the galleries collapsed and Pd nanoparticles were captured in a sandwichlike mesoporous architecture showing good accessibility to Pd nanoparticles. On the basis of X-ray photoelectron spectroscopy (XPS) and CO diffuse reflectance Fourier transform infrared spectroscopy (DRIFTS) Pd was found to be in a partially oxidized state, while a reduced Ti species indicated an electronic interaction between nanoparticles and nanosheets. The close contact of titanate sandwiching Pd nanoparticles, moreover, allows for the donation of a lattice oxygen to the noble metal (inverse spillover). Due to the metal–support interactions of this peculiar support, the catalyst exhibited the oxidation of CO with a turnover frequency as high as 0.17 s^–1^ at a temperature of 100 °C.

## 1 Introduction

Not only are supporting materials important to disperse and stabilize catalytically active nanoparticles but also extensive research gave convincing evidence for an active role of the support in the catalytic processes.^1^ Charge transfer between a (noble) metal and the support modifies the electronic structure and thus modulates the interaction with adsorbate molecules, a phenomenon referred to as an electronic metal–support interaction (EMSI).^2−6^ Furthermore, it was shown that catalytic reactions often occur at the perimeter between the support and metal, including spillover phenomena.^7,8^ Especially, model catalysts fabricated by deposition of ultrathin layers of oxides on atomically flat metal surfaces by means of vapor deposition have attracted much interest.^9−12^ These very defined model structures allowed systematic studies that led to fundamental insight into the catalytic performance. For instance, the metal work function can be significantly altered by the ultrathin oxide layer due to dipole effects arising from compression or charge transfer.^13−15^ Along this line, the adsorption behavior of H_2_ on a TiO_2_ monolayer with a lepidocrocite structure deposited onto Pt(111) and Ag(100) was computationally investigated.^16^ Charge transfer from Ag to the oxide was more pronounced than for Pt, and the accumulation of negative charge on the oxide disfavored the adsorption of H_2_. Furthermore, when the noble metal is only partially covered by the oxide layer, very reactive kinks between metal and oxide islands can be created.^17^ Even though such model systems are most helpful to understand fundamental mechanisms, transferring this knowledge to the bulk scale remains challenging.^18^

Recently, on application of a nematic phase of single sub-nanometer thick and negatively charged hectorite nanosheets,^19,20^ the transfer to bulk architectures was accomplished for Pd.^21^ Sandwiching Pd nanoparticles between two highly negatively charged hectorite sheets resulted in a positive charge on the Pd. This in turn improved the catalytic performance for the oxidation of carbon monoxide (CO) in comparison to the same nanoparticles deposited on a conventional support such as γ-Al_2_O_3_.

**Scheme 1:**
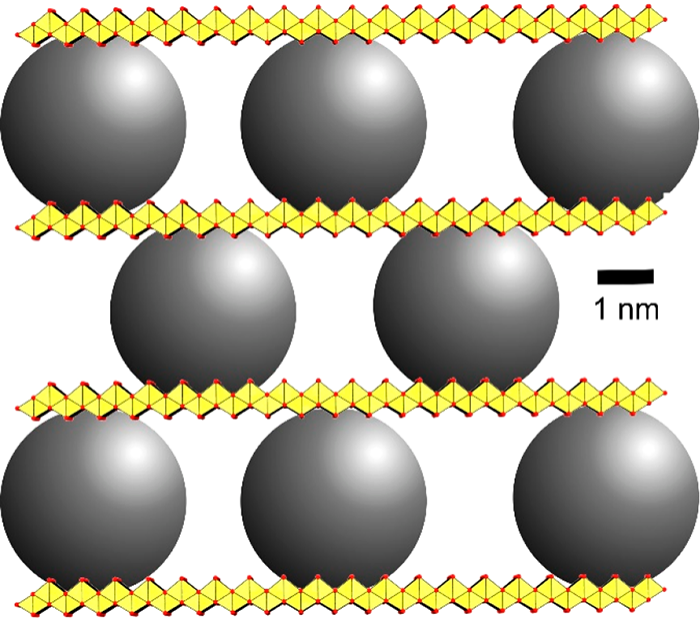
Sketch of the Porous Catalyst Structure Where Pd Nanoparticles Are Sandwiched between Adjacent L-Titanate Nanosheets

Nematic phases of coplanar nanosheets are also known for some transition-metal oxides. For instance, two-dimensional (2D) layered lepidocrocite-type titanates with a nominal formula of A_*x*_Ti_2–*y*_O_4_M_*y*_ (A = K^+^, Cs^+^, Rb^+^, M = Li^+^; vacancy; *x* = 0.7–0.8; abbreviated as L-titanate)^22,23^ appear promising in the context sketched above. These can be converted into a protonated form by acid treatment. Due to Ti^4+^ vacancies in the lepidocrocite sheet, they possess a permanent negative layer charge and, similarly to hectorite, nematic phases of single nanosheets are obtained by repulsive osmotic swelling.^24^ Due to the high layer charge, these nanosheets repel each other, which forces the nanosheets to adopt a cofacial orientation even at high dilutions, resulting in a liquid crystalline nematic phase showing structural colors.^25^ At high dilutions (typically 2 g L^–1^), the nanosheet separation is sufficient (typically 60 nm) to grant access for nanoparticles to the gallery between the nanosheets, creating a structure as sketched in Scheme 1.

For the oxidation of CO, redox-active supports that offer oxygen storage capacity, such as Co_3_O_4_, CeO_2_, and TiO_2_, are often used. A lattice oxygen of the support can then be donated to the oxide/metal perimeter, facilitating the oxidation reaction.^4,26,27^ The redox-active metal of the oxide support is reduced, while a vacancy in the oxide sublattice is left behind. In a later step of the catalytic cycle this vacancy is refilled by O_2_ from the gas phase. This mechanism is called the Mars–van Krevelen-mechanism. In the case of CO oxidation, this bypasses the CO poisoning at low temperatures that occurs on noble metals following the Langmuir–Hinshelwood mechanism, where CO and O_2_ competitively adsorb on the metal surface.^28−30^ Model catalysts proved that a direct relationship between the metal/support perimeter and activity existed and thus a maximized perimeter is desirable.^31^ A sandwichlike fixation of the Pd metal as sketched in Scheme 1 would furthermore lead to an extended perimeter to the support and thus increase the catalytic activity in comparison to a nanoparticle only in contact with a support from one direction.

Here, we report the intercalation-like heteroassembly of positively charged Pd nanoparticles and negatively charged nanosheets of L-titanate. The architectures of Pd supported on/sandwiched between L-titanates, mimic the model catalysts of ultrathin layers deposited on metal surfaces. The resulting catalyst was highly active in the oxidation of CO.

## 2 Results and Discussion

### 2.1 Synthesis and Characterization of L-titanate@Pd@L-titanate

H_1.07_Ti_1.73_O_4_·H_2_O (H^+^-L-titanate) was synthesized according to a published procedure via the solid-state synthesis of K_0.8_Ti_1.73_Li_0.27_O_4_ followed by an HCl treatment.^23^ To obtain a nematic phase, interlayer H^+^ was then exchanged for TBA^+^ (tetrabutylammonium) with a stoichiometric H^+^/TBA^+^ ratio of 1. The solid content of (TBA)_1.07_Ti_1.73_O_4_·H_2_O was 2 g L^–1^.^24^ Upon mechanical shaking a nematic phase was obtained with individual nanosheets separated to 59 nm according to small-angle X-ray scattering (SAXS; Figure S1). As the nematic phase of the titanate nanosheets was only stable at pH ≥10, Pd nanoparticles were required to carry a positive surface (ζ) potential at this pH. Therefore, 4-dimethylaminopyridine (DMAP) was applied as the capping ligand for the Pd nanoparticles, which in turn were synthesized by reduction of Na_2_[PdCl_4_] with NaBH_4_.^32^ The as-synthesized nanoparticles showed a narrow size distribution of 3.4 ± 0.4 nm, as determined by transmission electron microscopy (TEM; Figure S2a). According to dynamic light scattering (DLS), the nanoparticles were stable in aqueous dispersions at pH 10 with a hydrodynamic diameter of 4.5 ± 1.3 nm (Figure S2b) and a ζ potential of +28 mV, as determined by an electrophoretic measurement. The ζ potential of L-titanate was −39 mV at pH 10.

The Pd nanoparticles were added as a 0.1 wt % dispersion to the nematic phase of L-titanate under vigorous stirring, whereupon flocculation of the oppositely charged nano-objects occurred within 30 s. Elemental analysis (CHN) after repeated washing cycles showed that 5.77 wt % C and 0.44 wt % N remained in the catalyst (Table S1). This C/N ratio of 13.1 was much higher than for DMAP but was close to the expected value of 13.7 for TBA^+^, suggesting that ∼20% of the cation exchange capacity of the TBA^+^ remained in the structure after washing. Calcination in an air atmosphere for 5 h at 500 °C removed the residual organic content, as cross-checked by CHN analysis (Table S1). Furthermore, Fourier transform infrared spectroscpy (FTIR) was applied to detect possible OH groups left in the catalyst (Figure S3). No bands at around 3300 cm^–1^ or at 1641 cm^–1^ corresponding to the stretching and bending vibrations of H_2_O and H_3_O^+^ between the nanosheets were observed.^22,33^ Furthermore, no band between 950 and 1000 cm^–1^ became apparent, which would indicate Ti–OH groups formed by calcination and concomitant removal of interlayer water.^34^

**Figure 1:**
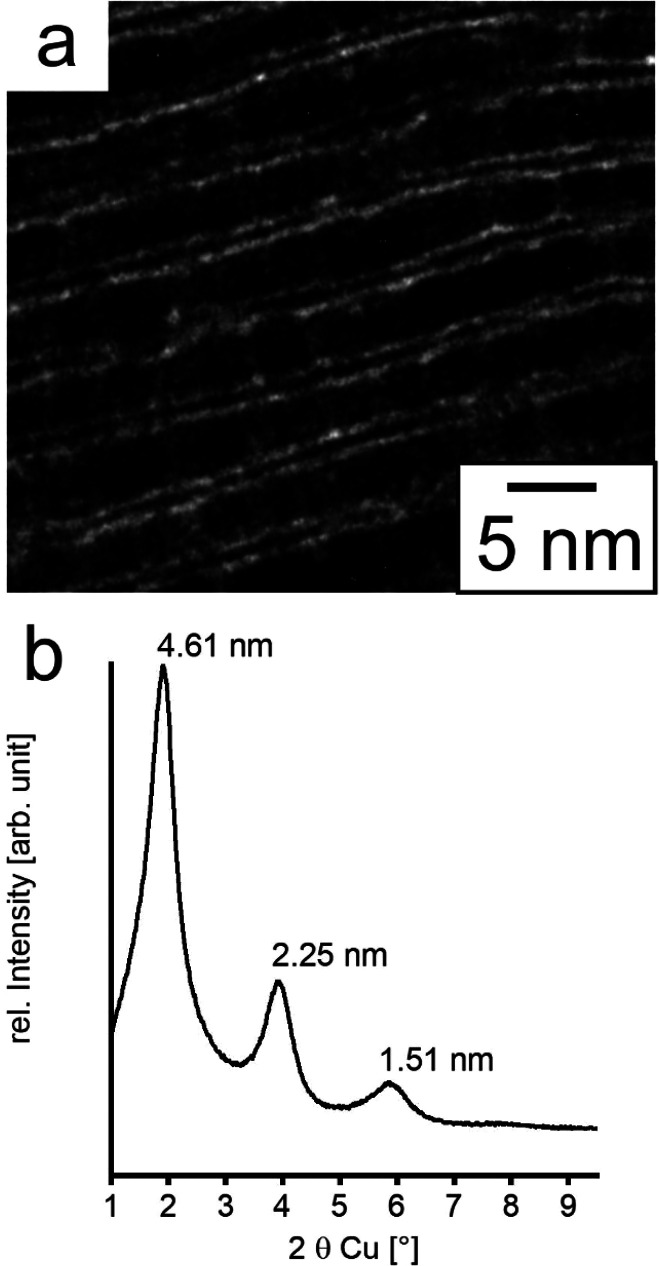
Evaluation of the lamellar structure of L-titanate@Pd@L-titanate: (a) TEM image of cross sections showing Pd nanoparticles confined between adjacent L-titanate nanosheets; (b) PXRD showing a series of basal reflections with a *d* value corresponding to the sum of the average diameter of the Pd nanoparticles and the thickness of single L-titanate nanosheets.

Upon calcination at 500 °C Pd was oxidized to PdO, which could easily be reduced back to metallic Pd under a flow of H_2_ (10% in N_2_) at 200 °C (Figure S4). The loading of Pd in the catalyst after calcination was as high as 49 wt %, as determined by scanning electron microscopy combined with energy dispersive X-ray spectroscopy (SEM-EDS). Furthermore, elemental mapping showed a homogeneous distribution of Pd (Figure S5). Upon flocculation the nanoparticles were trapped between the nanosheets, reflecting a support from two sides and creating a lamellar structure that was preserved after the calcination and successive reduction step (Figure 1a). Adjacent layers of nanoparticles were separated by one nanosheet of approximately 0.75 nm thickness. Thus, an architecture as sketched in Scheme 1 was obtained where oxide layer covered nanoparticles were stacked upon each other. The nanoparticles were not densely packed, as indicated by a grayscale analysis (Figure S6). Moreover, in this architecture the nanoparticles were in contact with the layered oxide from the top and bottom, which increases the metal/support perimeter area in comparison to nanoparticles having contact only from one direction.

Due to the sandwichlike confinement, the nanoparticles were stabilized against Ostwald ripening and preserved a size of 3.8 ± 0.6 nm during calcination and reduction. The high one-dimensional order along the stacking direction as already indicated by the TEM image was confirmed on the bulk scale by powder X-ray diffraction (PXRD) showing a series of basal reflections with a periodicity of 4.61 nm (Figure 1b). The basal spacing reflects the distance between adjacent nanosheets and is well in agreement with the expected value based on the sum of the average size of a Pd nanoparticle (3.8 nm) and the thickness of a single L-titanate nanosheet (0.75 nm).

Furthermore, in transmission mode, *hk* bands were observed at 25.2, 47.9, and 62.1° 2θ, indicating that the two-dimensional structures of L-titanate nanosheets^35^ were also preserved during preparation (Figure S4). This is in contrast to TBA^+^ intercalated L-titanate that readily undergoes a phase transition to anatase when it is heated to 500 °C (Figure S7), indicating that the separation of the nanosheets by Pd nanoparticles stabilizes the layered structure.^36^ When the nanosheets are kept at a 3.8 nm distance by intercalated Pd nanoparticles, this phase transition did not commence before 750 °C. This thermal stabilization is in line with observations that for single nanosheets of Ti_0.91_O_2_^0.36–^ the onset of the phase transition was as high as 800 °C and rapidly decreased to 400 °C when six layers were stacked in close contact.^37^

Ar physisorption of L-titanate@Pd@L-titanate gave a type IV(a) isotherm with H2(b) hysteresis (Figure S8 and Table 1), which was attributed to a mesoporous structure.^38^ The surface area on application of the BET equation was calculated to be 155 m^2^ g^–1^ and a median pore size of 6.5 nm was derived by applying the BJH method. The accessible metal dispersion was 19%, which expectedly was lower than for free-floating 3.8 nm Pd nanoparticles (29%), since sandwiching by the support covers a certain part of the surface (Table 1). Physisorption and chemisorption measurements both indicate that the nanoparticles were not densly packed, but mesopores between the nanoparticles make the Pd surface accessible.

**Table 1:** Surface Areas, Nanoparticle Size, And Catalytic Properties of L-titanate@Pd@L-titanate and of Reference Catalysts after Calcination at 500 °C and Reduction at 200 °C

sample	*S*_BET_ (m^2^ g^–1^)^a^	pore *d*_50_ (nm)^b^	*S*_Pd_ (m^2^ g^–1^)^c^	dispersion (%)^c^	core size of Pd (nm)^d^	*T*_50_ (°C)	*E*_A_ (kJ mol^–1^)
L-titanate@Pd@L-titanate	155	6.5	42	19	3.8 ± 0.6	86	38
Pd_ext_@P25	32		0.8	18	3.8 ± 0.8	148	48
Pd_ext_@Al_2_O_3_	156	7.6	1.0	23	3.6 ± 0.7	183	64

a Determined by Ar physisorption at 87 K and evaluated with the BET method.

b Determined by the BJH method.

c Determined by the CO chemisorption double isotherm method.

d Determined by TEM.

As was already mentioned, deposition of ultrathin nanosheets is expected to have a significant effect on the electronic properties of a (noble) metal. XP spectra of the Pd 3d region were recorded to probe the potential influence of the special architecture as sketched in Scheme 1 on Pd. For comparison, the same Pd nanoparticles used for the fabrication of L-titanate@Pd@L-titanate were also deposited with 1 wt % loading on commercial supports having a slightly lower ζ potential such as mesoporous γ-Al_2_O_3_ (Pd_ext_@Al_2_O_3_, −20 mV) and Degussa P25 (mixture of anatase and rutile, Pd_ext_@P25, −27 mV) (Table 1).

**Figure 2:**
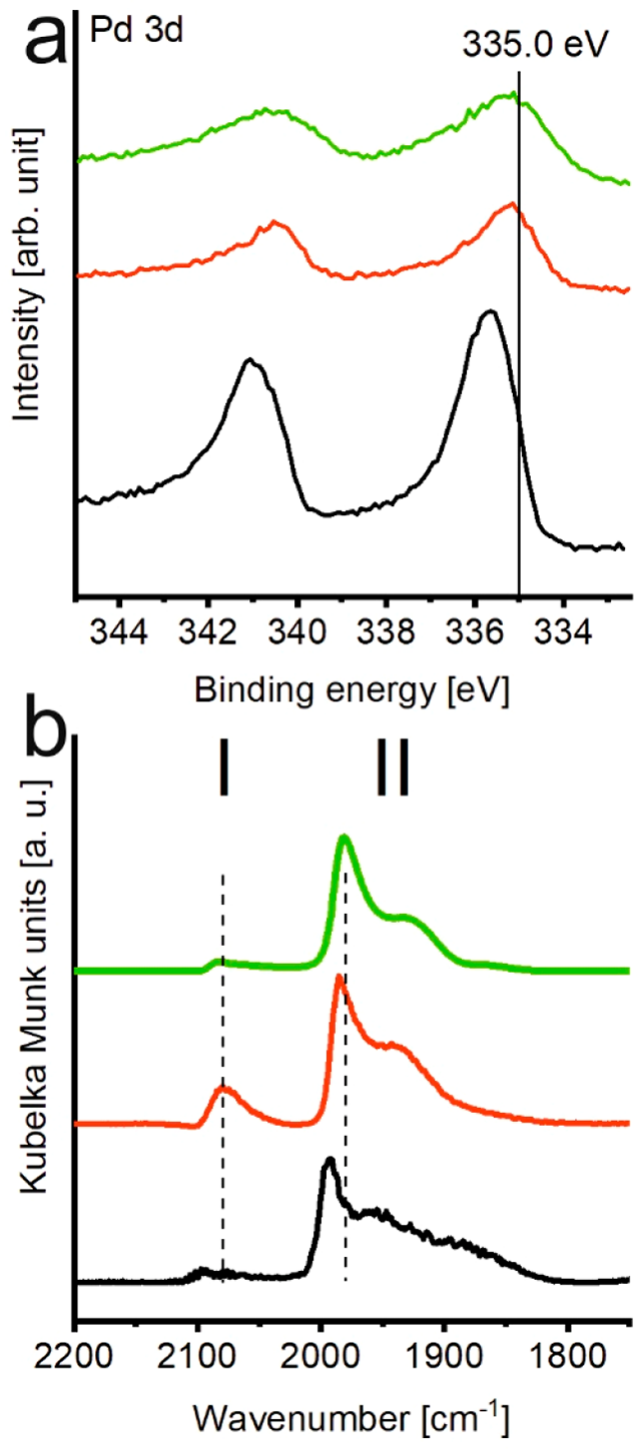
Evaluation of the surface oxidation state of Pd nanoparticles of L-titanate@Pd@L-titanate (black), Pd_ext_@P25 (red), and Pd_ext_@Al_2_O_3_ (green): (a) XP spectra of the Pd 3d region; (b) CO DRIFT spectra recorded at room temperature after saturation of the surface at 60 mbar of CO partial pressure followed by outgassing to 2 mbar.

**Figure 3:**
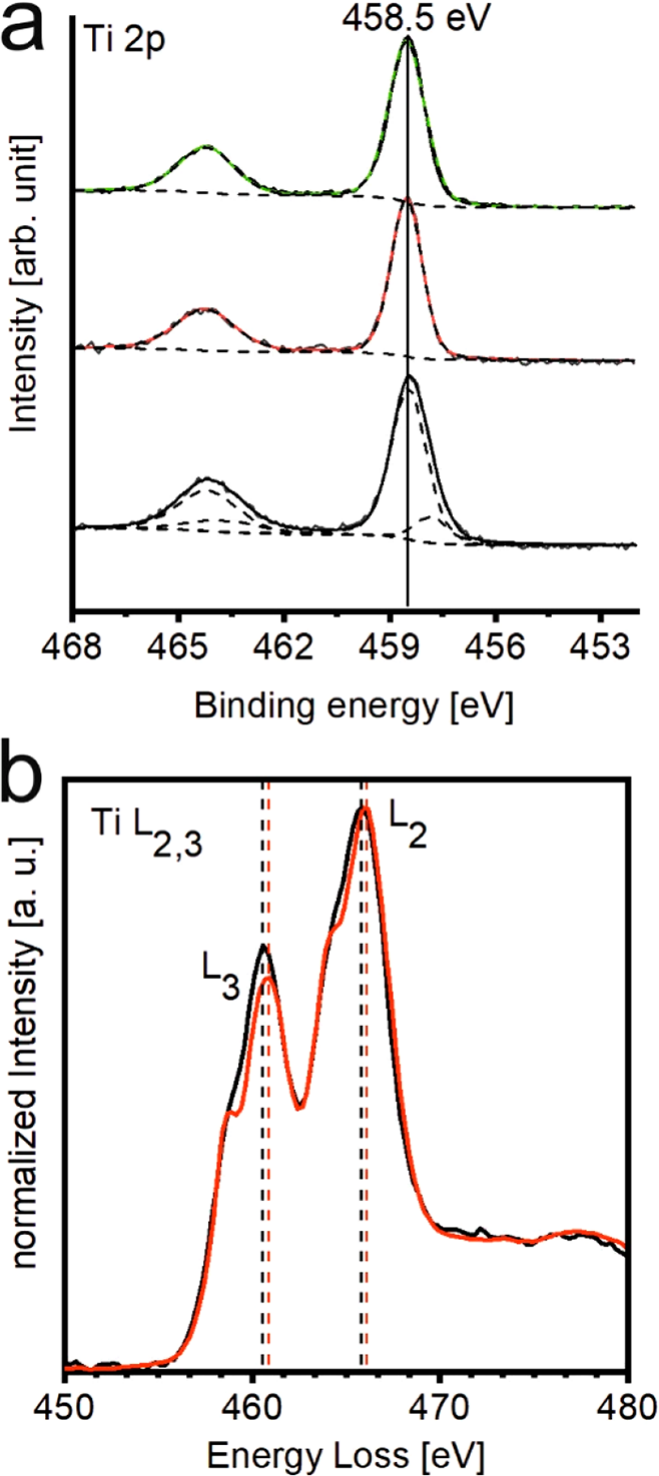
Evaluation of the charge state of Ti. (a) XP spectra of the Ti 2p region of L-titanate@Pd@L-titanate (black), H^+^-L-titanate (red), and Pd_ext_@P25 (green). The dashed lines are the peaks derived from deconvolution, and the colored lines are the overall fitted spectra. (b) EEL spectra at the Ti L_2,3_ edge of L-titanate@Pd@L-titanate (black) and H^+^-L-titanate (red), normalized to L_2_ maximum intensity.

Pd 3d spectra showed asymmetric signals of a spin orbit doublet with a splitting energy of 5.26 eV (Figure 2a). Asymmetric signals are derived from the high density of states of Pd at the Fermi level. The Pd 3d_5/2_ signal of L-titanate@Pd@L-titanate was found at a binding energy (BE) of 335.6 eV, which was significantly shifted from the 335.0 eV reported for bulk Pd.^39^ BEs of Pd nanoparticles deposited on TiO_2_ range from 334.6 to 335.5 eV.^40−44^ These reported shifts in comparison to the value of bulk Pd might be ascribed to electronic metal–support interactions. Along this line, the shift to higher BE observed for L-titanate@Pd@L-titanate would indicate a slightly positively charged species of Pd^δ+^(δ < 1).^45,46^ A shift to higher BE of small metal nanoparticles can, however, also originate from final state effects or an ill-defined reference level. The BE of the two reference catalysts Pd_ext_@Al_2_O_3_ and Pd_ext_@P25 were found at 335.1 and 335.3 eV, respectively. As these are comprised of identical nanoparticles, this indicates that the observed BE shift is indeed an initial state effect, rather than a final state effect. Moreover, the Ti 2p_3/2_ signal of L-titanate@Pd@L-titanate is found at 458.5 eV, matching the literature value for pristine L-titanate (Figure 3a).^47^ If the reference level would have been ill-defined, this peak should have been shifted to higher BE as well. The smaller shifts observed for the same Pd nanoparticles deposited on the reference supports therefore suggested a stronger interaction between the Pd nanoparticles sandwiched in L-titanate.

Additionally, diffuse reflectance infrared Fourier transform spectroscopy (DRIFTS) of chemisorbed CO was applied to probe the oxidation state of surface Pd atoms. When the CO partial pressure was increased to 60 mbar, signals experienced a shift to higher wavenumbers due to dipolar coupling caused by increasing surface coverage (Figure S9). A closer look at the DRIFT spectra recorded for samples that were outgassed to 2 mbar after having been saturated at a partial pressure of 60 mbar of CO yielded information on the electronic surface structure of Pd (Figure 2b) when they were compared with the reference samples. Two regions were observed for the C–O stretching vibration. In the case of Pd_ext_@Al_2_O_3_, the first region I with a peak centered around 2080 cm^–1^ was ascribed to linearly bound CO to corners (Figure S9d).^48,49^ The second and much broader region II is the superposition of several bands of CO bridge bound or 3-fold bound on different planes of Pd.^50,51^ At the same equilibrium pressure of CO (2 mbar) the DRIFT spectrum of L-titanate@Pd@L-titanate displayed the top band in region I with a peak centered at about 2090 cm^–1^. Furthermore, it appeared that the features of the second region also shifted by about 10–15 cm^–1^ to higher wavenumbers. As the same nanoparticles were used to fabricate both materials, the shift to higher wavenumbers may be attributed to weaker back-donation of electrons from the Pd surface to the antibonding CO 2π* orbital. This strengthens the C–O bond and consequently shifts the stretching vibration to higher wavenumbers. A partial positively charged Pd surface supported on L-titanate@Pd@L-titanate as suggested by XPS data might be the reason for reduced back-bonding.^50^ The DRIFTS bands for the titania-supported reference (Pd_ext_@P25) are shifted in the direction of L-titanate@Pd@L-titanate in comparison to Pd_ext_@Al_2_O_3_ but to a much lesser extent (e.g. 2083 cm^–1^ for the top band). This is in line with the smaller positivation of Pd by the P25 support, as also corroborated by the XPS data.

The electron deficiency of Pd nanoparticles in L-titanate@Pd@L-titanate might actually arise from two factors. The first is the need to balance the permanent negative layer charge of L-titanate nanosheets. Furthermore, an additional contribution might originate from electronic interactions between the metal and the oxide that were reported between noble metals and noncharged TiO_2_.^16,46^ To probe the latter, the Ti 2p regions in the XPS of L-titanate@Pd@L-titanate and of H^+^-L-titanate before intercalation of the nanoparticles were compared (Figure 3a). The Ti 2p_3/2_ signal of H^+^-L-titanate was found at a BE of 458.5 eV, which can be ascribed to Ti^4+^.^47^ For L-titanate@Pd@L-titanate the Ti 2p_3/2_ signal was significantly broadened and deconvolution of the signal gave an additional peak at 457.8 eV that might be attributed to Ti^δ+^ (δ < 4) sites. Additionally, electron energy loss spectroscopy (EELS) at the Ti L_2,3_ edge was conducted to further corroborate the existence of a slightly reduced Ti species (Figure 3b). The Ti L_2,3_ edge corresponds to the transition of Ti 2p_1/2_ and Ti 2p_3/2_ electrons into unoccupied states.^52^ The signal position and shape for H^+^-L-titanate were in agreement with literature data.^53^ The EEL spectrum of L-titanate@Pd@L-titanate was noticeably different from that of H^+^-L-titanate and supports the postulation of a reduced Ti species due to the presence of Pd. The white lines of Ti L_2_ and Ti L_3_ of L-titanate@Pd@L-titanate were shifted by about 0.35 eV to lower values in comparison to H^+^-L-titanate, as expected for reduced Ti species.^52^ While this shift is small and is on the scale of the energy dispersion of these spectra (0.25 eV/channel), there are more spectral features that point toward reduced titanium: in the O K edge (Figure S10), the ionization edge is shifted to higher energies, which is in agreement with reduced Ti species.^54^ Additionally, the induced crystal field splitting observed at the O K edge has been shown to decrease from Ti^4+^ to more reduced species.^54−56^ This effect is visible in the O K edge, as H^+^-L-titanate features a splitting of 2.2 eV, whereas this decreases to 1.8 eV upon introduction of Pd. This difference in crystal field can also be seen in the Ti L edge, where both the L_3_ and L_2_ edges of Ti^4+^ are known to feature doublets due to this splitting.^56^ The empty d band is split by a crystal field, and the resulting e_g_ and t_2g_ states then become part of the unoccupied conduction bands. The degree to which the d states are then filled up on partial charge transfer changes the intensities in the spectra and influences other effects that spectrally overlap with crystal-field splitting, such as exchange splitting, which results in different (apparent) e_g_/t_2g_ ratios.^55^ Finally, the L_2_/L_3_ intensity ratios of Ti decrease with a decreasing average oxidation state of Ti.^52,57^ Indeed, the ratio for L-titanate@Pd@L-titanate is 9% smaller than that for H^+^-L-titanate. All these EELS features are in line with the shifts in BE observed by XPS and indicated that the average oxidation number of Ti is slightly lowered after intercalation of Pd nanoparticles. In summary, XPS data for Pd and Ti, EELS for Ti, and CO-DRIFTS all gave significant evidence that electronic interactions between Pd nanoparticles and L-titanate nanosheets exist and might in turn influence the catalytic performance.

### 2.2 Catalysis

**Figure 4:**
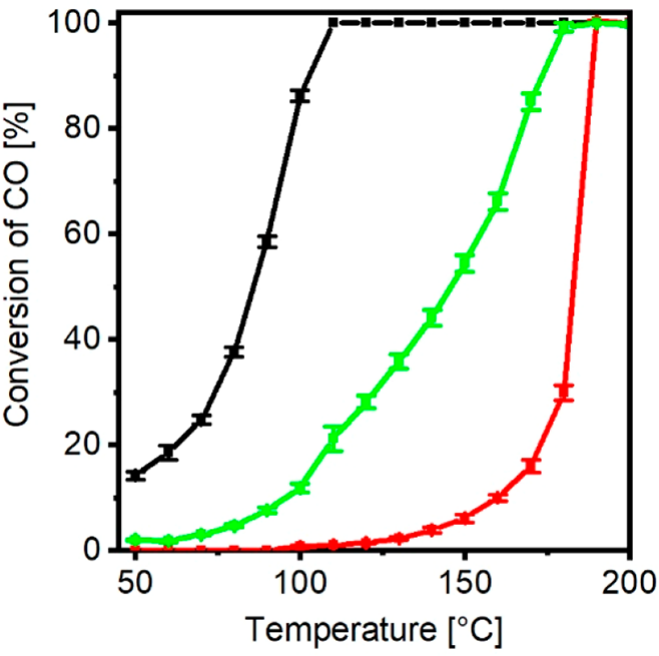
Light-off curves of L-titanate@Pd@L-titanate (black), Pd_ext_@P25 (green), and Pd_ext_@Al_2_O_3_ (red). Conditions: 1 mg of Pd per catalysis; 50 mL/min (1 vol % CO, 1 vol % O_2_, balanced in N_2_).

CO oxidation is one of the most frequently studied heterogeneous catalytic reactions due to its importance for exhaust gas purification or reduction of industrial emissions. Furthermore, as adsorbed CO is very sensitive to electronic influences of the support, the CO oxidation is ideally suited as a model reaction to probe for the potential influence of the special support architecture of L-titanate@Pd@L-titanate.^58,59^ For the catalytic tests, the amount of catalyst was chosen to involve 1 mg of Pd in a fixed bed reactor with a feed gas stream of 50 mL/min (1 vol % CO, 1 vol % O_2_, balanced by N_2_). All catalysts were pretreated under the same conditions to ensure comparability (500 °C in an air atmosphere for 5 h, followed by H_2_ treatment at 200 °C for 2 h). Three light-off curves were measured for each catalyst, and the third curve is presented in Figure 4.

L-titanate@Pd@L-titanate exhibited a high performance at low temperatures with temperatures for 50% conversion (*T*_50_) and for full conversion (*T*_100_) as low as 86 and 110 °C, respectively (Figure 4). The same Pd nanoparticles deposited on γ-Al_2_O_3_ (Pd_ext_@Al_2_O_3_) were inferior by far with *T*_50_ and *T*_100_ values of 183 and 190 °C, respectively. The activation energy *E*_A_ of 64 kJ mol^–1^ for Pd_ext_@Al_2_O_3_ as derived from the Arrhenius plot (determined below conversions of 10%, Figure S11) matched reported values.^58^ The low *E*_A_ of 38 kJ mol^–1^ observed for L-titanate@Pd@L-titanate is in the range typically found for catalysts that follow a Mars–van Krevelen type reaction mechanism.^60^

The shape of the light-off curve of Pd_ext_@Al_2_O_3_ showed a sharp increase in conversion at higher temperatures. CO oxidation for Pd@Al_2_O_3_ catalysts follows the Langmuir–Hinshelwood mechanism.^28^ CO and O_2_ compete for adsorption at the Pd surface. CO binds strongly to the Pd surface at lower temperature and O_2_ can only coadsorb at higher temperatures. As the reaction is highly exothermic, the conversion normally increases sharply after light-off.

**Figure 5:**
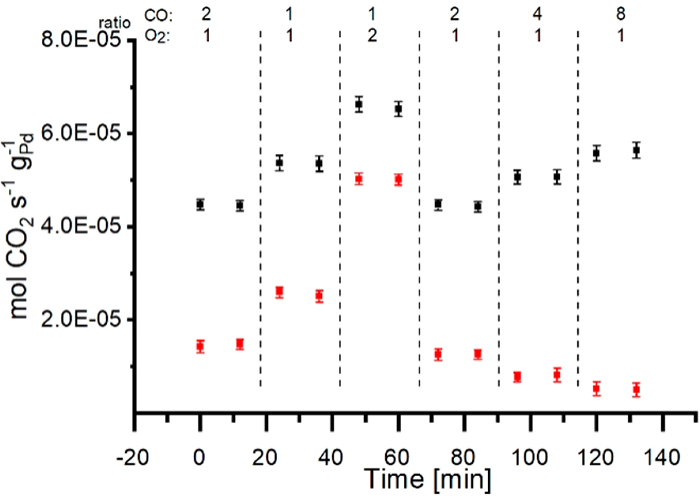
Kinetic rates of L-titanate@Pd@L-titanate at 70 °C (black) and Pd_ext_@Al_2_O_3_ at 130 °C (red) at different partial pressures of CO and O_2_.

As was recently reported,^21^ sandwiching of Pd nanoparticles between the negatively charged nanosheets of the layered silicate hectorite (Hec@Pd65@Hec) decreased the *T*_50_ value from 191 to 145 °C for the oxidation of CO in comparison to the same nanoparticles deposited on γ-Al_2_O_3_. For Hec@Pd65@Hec a positive surface charge was observed by shifts in the XPS Pd 3d region and CO-DRIFTS. This positive surface charge was attributed not only to balancing of the negative layer charge but also to electronic interactions between the silicate nanosheet and Pd. As the CO reaction followed the Langmuir–Hinshelwood mechanism that requires adsorption of both CO and O_2_ to the noble-metal surface, the positive surface charge of Pd decreased the adsorption energy of CO, which allowed O_2_ to already coadsorb at lower temperatures. Here, negatively charged nanosheets of a different composition, but similar thickness and charge density, were applied. These nanosheets demonstrated a similar influence on the surface charge of Pd and the electronic interaction between the support and the metal (Figures 2 and 3). However, on application of the same catalytic conditions, the performance of L-titanate@Pd@L-titanate was much higher at low temperatures (*T*_50_ value of 86 °C). This implies that L-titanate must have some additional influence on the catalytic activity. A possible explanation is that L-titanate might be able to offer oxygen at the nanoparticle/oxide perimeter that omits the necessity of oxygen adsorption directly to the Pd surface. This would allow the reaction to already occur at lower temperatures. Kinetic measurements (Figure 5 and Tables S2 and S3) were applied to study the influence of the CO and O_2_ partial pressures on the reaction rates. Therefore, the composition of the reactant flow was varied, while the temperature was kept constant at 70 and 130 °C for L-titanate@Pd@L-titanate and Pd_ext_@Al_2_O_3_, respectively. Additional information about the procedure is given in the Supporting Information.

The reaction order with respect to the O_2_ partial pressure for Pd_ext_@Al_2_O_3_ was +0.91, which is consistent with the expected order of 1 for a Langmuir–Hinshelwood mechanism.^61,62^ In contrast, the reaction order with respect to the O_2_ partial pressure of L-titanate@Pd@L-titanate is +0.29. The low order is a hint that oxygen is provided from the supporting oxide nanosheets rather than from the gas phase.^28^ While oxygen donation is actually expected for oxides such as bulk anatase,^26,29^ it is still somewhat surprising that even sub-nanometer thick corrugated single layers of condensed octahedra are capable of coping with the structural defects caused by donating oxygen. Furthermore, the reaction order with respect to CO was +0.13 for L-titanate@Pd@L-titanate, while it was −0.67 for Pd_ext_@Al_2_O_3_. The negative order for the latter is expected for metallic Pd, as strongly binding CO poisons the surface. In contrast to this, the positive order observed for L-titanate@Pd@L-titanate indicated that this catalyst system does not suffer from CO poisoning at lower temperatures.

The *T*_50_ (148 °C) and *E*_A_ values (48 kJ mol^–1^) for Pd_ext_@P25 were much higher than for L-titanate@Pd@L-titanate. As the P25 support can also provide oxygen from its lattice, the crucial factor for the higher activity of L-titanate@Pd@L-titanate appears to be the special sandwich architecture and the advantageous electronic interaction with the anionic support. Another activity-enhancing factor is the interface area between the support and the metal, through which oxygen can be donated from the support to the metal. The activity of model catalysts of Pd nanoparticles deposited on CeO_2_ increased with the interface area between metal and support.^31^ Due to the special architecture of L-titanate@Pd@L-titanate the nanoparticles are in contact with the oxide from the top and bottom, creating a large boundary in comparison with nanoparticles solely supported on external surfaces.

As single-atom and small Pd cluster catalytic systems have shown a higher catalytic activity,^28^ the potential stabilization of such species might be an alternative explanation for the good performance of L-titanate@Pd@L-titanate. Since the preparation involved calcination at 500 °C followed be reduction, we regard it as highly unlikely that such small species could exist in L-titanate@Pd@L-titanate.

The sandwich architecture of L-titanate@Pd@L-titanate, moreover, inhibited catalyst deactivation. At 100 °C no significant reduction in the activity was observed after 72 h on stream (Figure S12a). Carbonate formation is reported to be one reason for catalyst deactivation,^2^ but for L-titanate@Pd@L-titanate this seems to be insignificant (Figure S12b). The average nanoparticle diameter was determined to be 3.9 ± 0.7 nm, which within experimental error was unchanged, demonstrating a hampered sintering of the nanoparticles. Furthermore, L-titanate@Pd@L-titanate was calcined at 700 °C for 40 h to probe the efficiency of the sandwich confinement to hamper Ostwald ripening under harsh conditions. The light-off curve after this treatment revealed a *T*_50_ value of 92 °C that was only slightly higher than the 86 °C observed for L-titanate@Pd@L-titanate after calcination at 500 °C (Figure S12c). This further demonstrated the good stability of L-titanate@Pd@L-titanate, making the catalyst promising for applications where the catalyst has to stand more demanding conditions.

## Conclusion

Ultrathin oxides have been demonstrated to alter the electronic structure of an underlying (noble) metal or create highly active perimeters on only partial coverage. This architecture can be mimicked by intercalation of positively charged metal nanoparticles between negatively charged nanosheets, as proven for L-titanate or previously for silicate nanosheets. Sandwiching Pd nanoparticles between negatively charged nanosheets triggers a partially oxidized state of the metal, as evidenced from an XPS shift of the Pd 3d region by +0.6 eV, and shifts of the C–O stretching bands of +10–20 cm^–1^, as derived from DRIFTS measurements. In contrast to the silicate nanosheets investigated previously, L-titanate nanosheets can additionally provide lattice oxygen, which further enhanced the performance (*T*_50_ value of 86 °C) in comparison to the silicate nanosheets (*T*_50_ value of 145 °C). Obviously, this special support architecture might also be attractive for other catalytic reactions such as methane combustion.^63,64^ The synthesis route via intercalation into nematic phases of anionic nanosheets is not restricted to Pd or to titanate nanosheets. Other liquid crystalline supports such as layered antimony phosphates^65,66^ will be explored in the future. Needless to say, the concept can also be extended to catalytically more attractive alloy nanoparticles.^67−70^

## Supporting Information Available

Experimental section including catalyst preparation and measurement techniques, SAXS of the nematic L-titanate phase, TEM image of Pd nanoparticles, elemental analysis of L-titanate@Pd@L-titanate, FTIR spectrum of L-titanate@Pd@L-titanate, XRD patterns for phase evaluation, elemental mapping, grayscale analysis of L-titanate@Pd@L-titanate, Ar isotherms, DRIFT spectra at various CO partial pressures, EEL spectra at O K edge, Arrhenius plots, and details about the kinetic investigation and stability test (PDF)
