# Supplementary material for: Enhancing the Catalytic Activity of Palladium Nanoparticles via Sandwich-Like Confinement by Thin Titanate Nanosheets
Source: ACS Catal. 2021 Feb 15;11(5):2754–62. doi: 10.1021/acscatal.1c00031 (PMC8016112; doi:10.1021/acscatal.1c00031)
Supplement: Supplementary file 1 — cs1c00031_si_001.pdf [file cs1c00031_si_001.pdf]

Supporting information

Enhancing the Catalytic Activity of Palladium Nanoparticles via Sandwich-Like Confinement by Thin Titanate Nanosheets

Kevin Ament[1], Daniel R. Wagner[1], Thomas Götsch[2], Takayuki Kikuchi[3], Jutta Kröhnert[2], Annette Trunschke[2], Thomas Lunkenbein[2], Takayoshi Sasaki[3]*, and Josef Breu[1]*

1 Bavarian Polymer Institute and Department of Chemistry, University of Bayreuth, 95447 Bayreuth (Germany)

E-mail: josef.breu@uni-bayreuth.de

2 Department of Inorganic Chemistry, Fritz-Haber-Institut der Max-Planck-Gesellschaft, Faradayweg 4-6, 14195 Berlin (Germany)

3 International Centre for Materials Nanoarchitectonics (WPI-MANA), National Institute for Materials Science (NIMS), 1-1 Namiki, Tsukuba, Ibaraki 305-0044 (Japan)

E-mail: sasaki.takayoshi@nims.go.jp

Table of contents

1 Experimental Procedures

2 Results and Discussions

2.1 Small angle X-ray scattering of a nematic titanate phase

2.2 Characterization of DMAP capped Pd nanoparticles

2.3 Elemental analysis of L-titanate@Pd@L-titanate

2.4 Phase evaluation of Pd in L-titanate@Pd@L-titanate catalyst

2.5 Elemental mapping over L-titanate@Pd@L-titanate

2.6 Grayscale analysis of L-titanate@Pd@L-titanate

2.7 Phase evaluation of L-titanate@Pd@L-titanate

2.8 Ar-Isotherms

2.9 DRIFT spectra at increasing CO pressure

2.10 EEL spectra at the O K edge

2.11 Arrhenius plots

2.12 Evaluation of the kinetic experiments

2.13 Long term stability of L-titanate@Pd@L-titanate

3 References

1 Experimental Procedures

Materials

$PdCl_2$ (99.999% Pd, Premion), NaCl, 4-Dimethylaminopyridine (DMAP), NaOH and $NaBH_4$ were purchased from Alfa Aesar. γ-Al_2O_3 with high surface area was purchased from Alfa Aesar. The pellets were grounded to a powder before use. Degussa P25 was purchased from Sigma Aldrich. The used water was of MilliQ quality (18.2 M☐).

Catalyst synthesis

Synthesis of Pd nanoparticles:

The synthesis of Pd nanoparticles was executed using a modified literature procedure.[1] Palladium(II) chloride (235 mg, 1.33 mmol) and Sodium chloride (155 mg, 2.66 mmol) were dissolved in 40 ml water and 4-Dimethylaminopyridine DMAP (833 mg, 6.82 mmol) in 80 mL water was added. After 20 min of stirring $NaBH_4$ (110 mg, 2.91 mmol) in 11 ml water was added dropwise under vigorous stirring resulting in a black dispersion. After 2 h the nanoparticle dispersion was dialyzed in 4 l water with water changes after 12 and 24 hours.

$K_{0.8}[Ti_{1.73}Li_{0.27}]O_4$ was synthesized via solid state synthesis and is described elsewhere.[2] The protonic form was obtained by stirring the solid powder in 1 M HCl solution at room temperature. The solution was replaced each day for 3 days. After this treatment the solid was filtered and washed with excess water to obtain $H_{1.07}Ti_{1.73}O_4 \cdot H_2O$. For delamination to 0.4 g protonic titanate an aqueous solution of TBAOH (10 %) was added to achieve a molar ratio TBA^+/H^+ of 1. The mixture was mechanically shaken for 7 days to achieve a nematic phase. The final solid content was 2 g/L.

Synthesis of L-titanate@Pd@L-titanate:

Both the aqueous particle and titanate dispersions were adjusted with NaOH resulting in a surface potential of the nanoparticles of 28 mV. The nematic titanate suspension was added rapidly to the nanoparticle dispersion under vigorous stirring. Visible flocculation appeared within 30 seconds. The black flocculate was separated from the supernatant by centrifugation, repeatedly washed and dried at 120 °C. Residual organics were removed by calcination at 500 °C for 5 h. Pd nanoparticles were regenerated under a flow of 30 mL/min (10 % H_2 in N_2) at 200 °C for 2 h.

Synthesis of Pd_{ext}@P25:

To a dispersion of Pd nanoparticles (1 mg/ml) Degussa P25 was added under stirring. The amount was chosen to obtain 1 wt% of Pd. After 24 h the resulting black solid was separated by centrifugation and washed several times. The catalyst was dried at 130 °C.

Synthesis of Pd_{ext}@Al_2O_3:

To a dispersion of Pd nanoparticles (1 mg/ml) was added γ-Al_2O_3 under stirring. The amount was chosen to obtain 1 wt% of Pd. The solvent was slowly removed under stirring at 80 °C. The catalyst was finally dried at 130 °C.

To obtain comparable conditions, all catalysts were calcined at 500 °C in flowing air for 5 h, followed by activation at 200 °C in a flow of H_2 (10 vol% in N_2) for 2 h.

Measurement and Characterization Techniques

Dynamic light scattering (DLS) and determination of ζ-potential were recorded on a Litesizer 500 (Anton-Paar).

CHN analysis was acquired with an Elementar Vario EL III.

Powder X-ray diffraction (PXRD) measurements were done using a STOE Stadi P diffractometer. Cu_{Ka1} radiation and a Mythen 1K silicon strip-detector were used. PXRD of traces of textured samples were recorded on a Bragg-Brentano type diffractometer (Empyrean, PANalytical) with nickel filter and $Cu_{K\alpha}$ radiation (λ = 1.54187 Å).

SAXS data were measured using a "Double Ganesha AIR"system (SAXSLAB,Denmark). The X-ray source of this laboratory-based system is a rotating anode (copper, MicroMax 007HF, Rigaku Corporation,Japan) providing a microfocused beam. The data is recorded by a position sensitive detector (PILATUS 300 K, Dectris)

Transmission electron microscopy (TEM) images were acquired using a JEOL JEM-2200FS (200 kV). For cross sectional TEM the powder embedded into a resin (EPO-TEK 301) and was cut with an Ar beam into thin slices using a Jeol Cryo Ion Slicer.

Scanning electron microscopy (SEM) images and energy dispersive spectroscopy (EDS) were recorded on a FEI Quanta FEG 250.

Photoelectron spectroscopy (XPS) was conducted on a PHI 5000 Versa Probe III fitted with an Al Kα excitation source and spectra were analyzed with Multipak software pack. Spectra were referenced to C 1s at 284.8 eV.

Electron energy loss spectroscopy (EELS) measurements were conducted using a double-corrected JEOL JEM-ARM200F microscope, operated at 200 kV and equipped with a Gatan GIF Quantum imaging filter with DualEELS capabilities. Plural scattering was removed from all spectra by Fourier ratio deconvolution.

Adsorption isothermes were recorded on a Quantachrome Autosorb-1 with Ar as adsorbate at 87 K. The isotherms were evaluated using Brunauer-Emmet-Teller (BET) method and pore size distribution was evaluated with BJH method. Metal surface was acquired with a Quantachrome Autosorb-1 with CO at 35 °C using the double isotherm method.

Diffuse reflectance infrared Fourier transform (DRIFT) spectra were collected on a Cary 680 FTIR spectrometer from Agilent equipped with a MCT detector and a Praying Mantis™ low temperature reaction cell from Harrick. Spectra were recorded at a spectral resolution of 2 cm−1 and accumulation of 1024 scans. Samples were dried at 130 °C under a flow of Ar (50 ml/min) over night and then reduced at 150 °C under a flow of H2 (10 vol%) for 1 h. To remove H2 the samples were evacuated at 150 °C for 30 min and then let allow to cool to room temperature. Spectra were taken at 300 K. CO isotherms were recorded by dosing CO at increasing equilibrium pressures ranging from 0.03 to 60 mbar. The spectra are presented in Kubelka–Munk units F(R∞) = (1 − R∞)2/2R∞

Catalysis

Catalytic tests were conducted in a fixed bed micro reactor with an internal diameter of 4 mm. The desired amount of catalyst was mixed with quartz to achieve an overall loading of 500 mg. The reactor was heated using a circular kiln. To record light-off curves the temperature was raised in 10 °C steps and the temperature was held for 12 min before analysis. A reactant mixture of 1 vol% CO, 1 vol% O_2 und 98 vol% nitrogen with a constant flow of 50 ml/min under atmospheric pressure was injected into the reactor. The composition of the gas mixture leaving the reactor was monitored using an Agilent 6890N gas chromatograph equipped with a 30 m GS CARBONPLOT column and a thermal conductivity detector. The catalysts were cylcled three times from 50 to 200 °C and the third light-off curve was recorded. Kinetic experiments and reaction rates were determined well below a conversion of 10 %.

2 Results and Discussion

2.1 Small angle X-ray scattering of a nematic L-titanate phase.

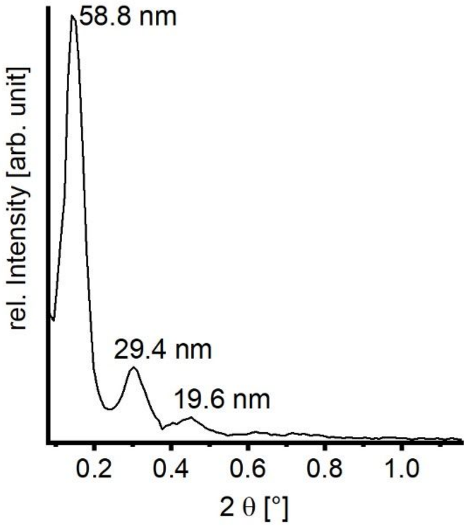

Figure S1. SAXS of a nematic L-titanate phase having a solid content of 2 g·L^{-1}.

2.2 Characterization of DMAP capped Pd nanoparticles

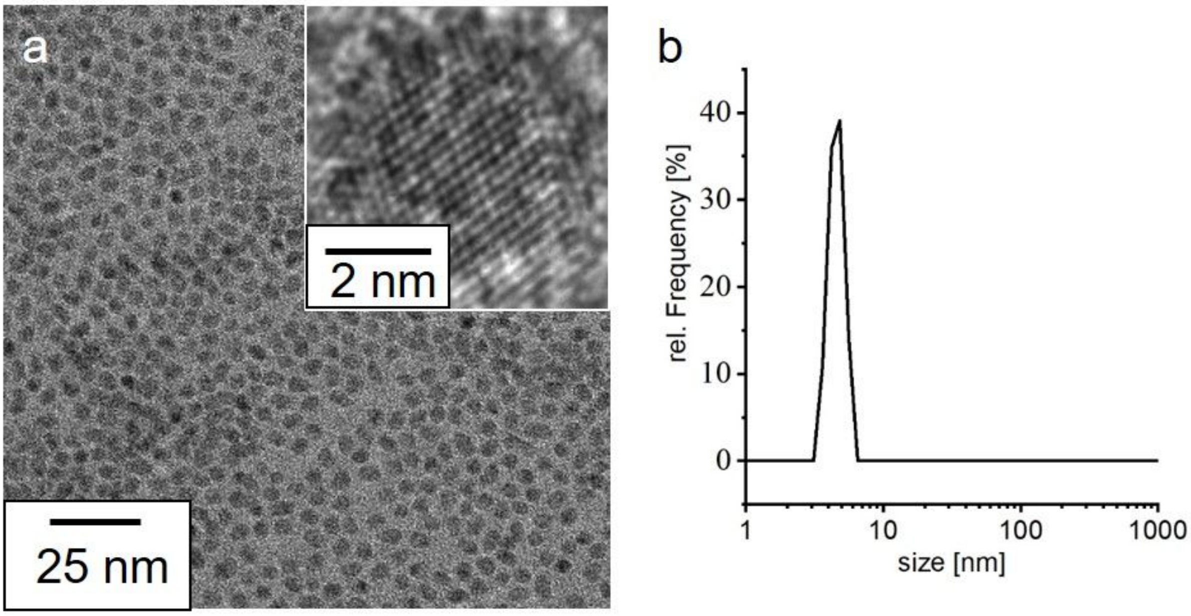

Figure S2. a) TEM image of as-synthesized Pd nanoparticles. Inset: High magnification image. b) Hydrodynamic diameter according to dynamic light scattering.

2.3 Elemental analysis of L-titanate@Pd@L-titanate

Table S1. Elemental composition of L-titanate@Pd@L-titanate

Sample	Fraction of C [wt%][a]	Fraction of N [wt%][a]	Fraction of H [wt%][a]	Fraction of Pd [wt%][b]
$(TBA^+/H^+)_{1.07}Ti_{1.73}O_4$	48.09	3.43	8.21	/
L-titanate@Pd@L-titanate	5.77	0.44	1.29	/[c]
L-titanate@Pd@L-titanate after 500 °C for 5 h	0.2	0.01	0	49.3

a determined by CHN analysis
b determined by SEM-EDS
c not determined

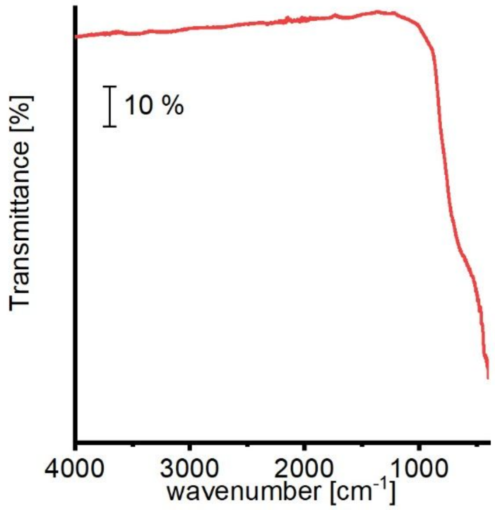

Figure S3. FTI spectrum of L-titanate@Pd@L-titanate.

2.4 Phase evaluation of Pd in L-titanate@Pd@L-titanate catalyst

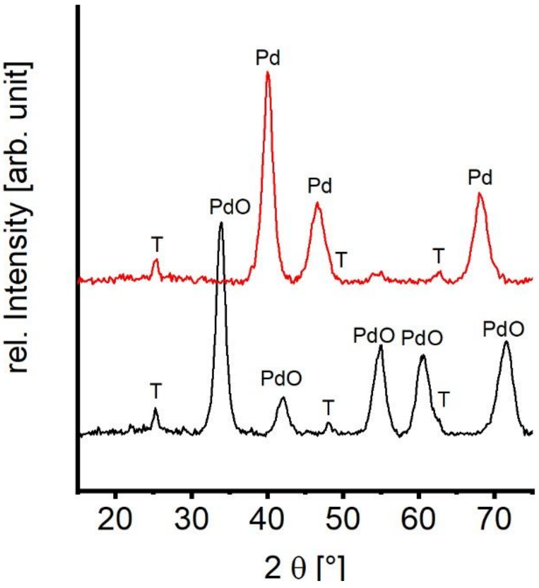

Figure S4. PXRD of L-titanate@Pd@L-titanate calcined for 5 h at 500 °C in air atmosphere (black) and after reduction at 200 °C for 2 h under a flow of H_2 (10 vol% in N_2) (red). T stands for reflections derived from the L-titanate nanosheets.[2]

2.5 Elemental mapping over L-titanate@Pd@L-titanate

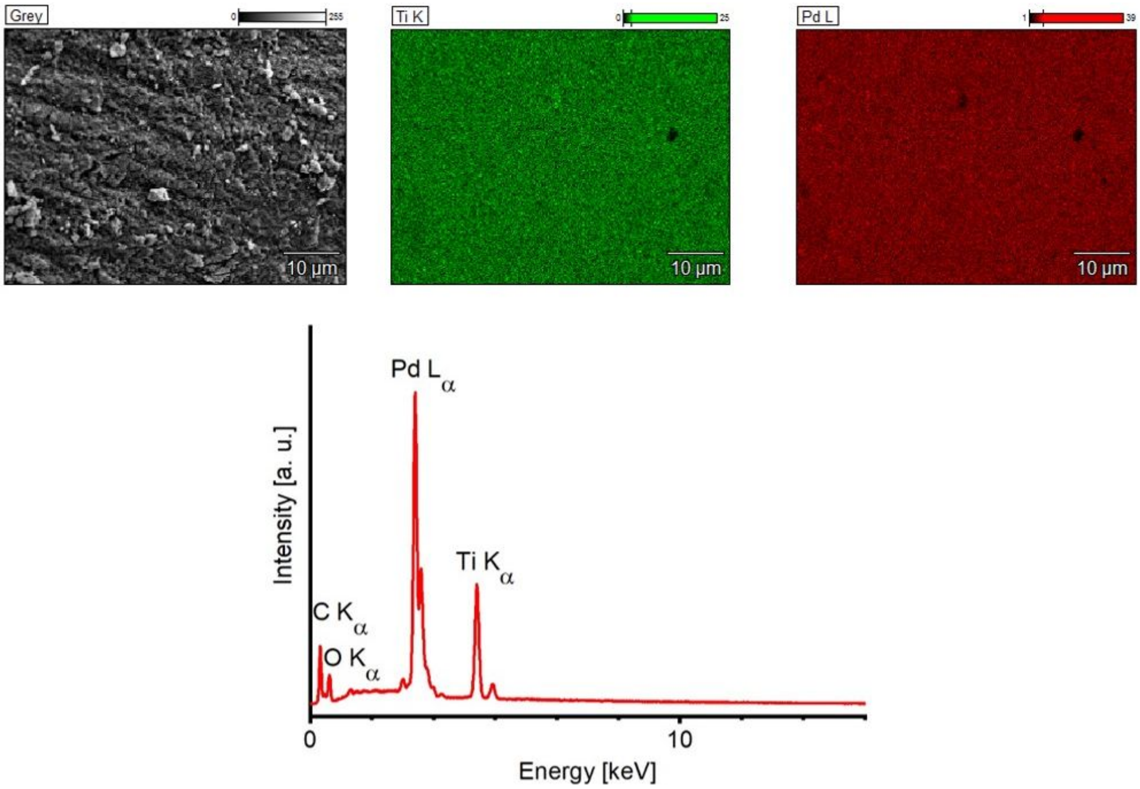

Figure S5. Elemental mapping of Ti and Pd showing a homogenous distribution of Pd and Ti over the tactoids and the corresponding spectrum.

2.6 Grayscale analysis of L-titanate@Pd@L-titanate

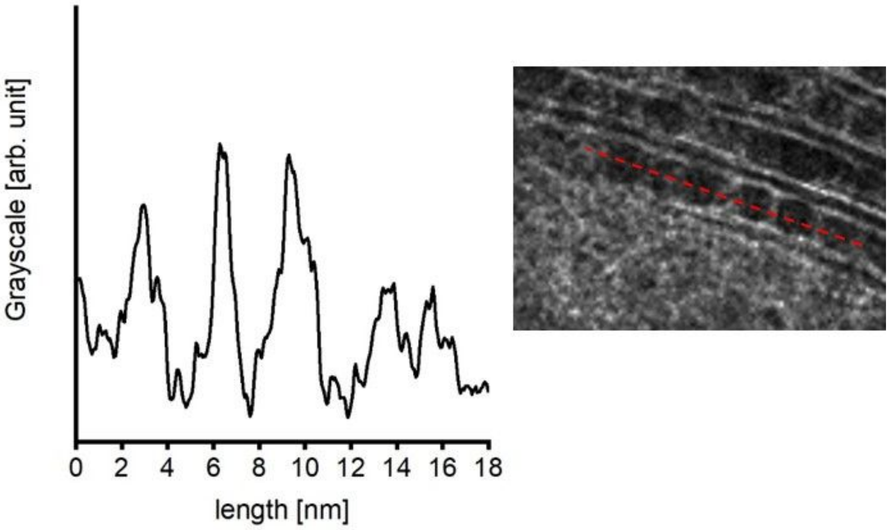

Figure S6. Grayscale analysis over a nanoparticle layer (red dotted line) between L-titanate nanosheets. The grayscale shows gaps between the nanoparticles proving a not densely packed assembly.

2.7 Phase evaluation of L-titanate@Pd@L-titanate

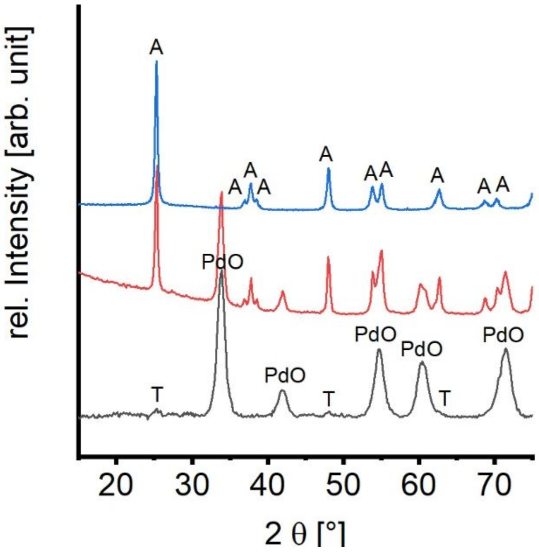

Figure S7. PXRD of L-titanate@Pd@L-titanate after calcination at 700 °C (black), calcination at 750 °C (red), and $(TBA/H)_{1.07}Ti_{1.73}O_4$ after calcination at 500 °C. T stands for reflections derived from the L-titanate nanosheets.[2] A stands for reflections from anatase phase. The PdO reflections remain very broad even after treatment at 700 °C. Only above 700 °C the network breaks down which allows the nanoparticles to grow (narrower reflections) and the L-titanate transforms to the anatase phase.

2.8 Ar-Isotherms

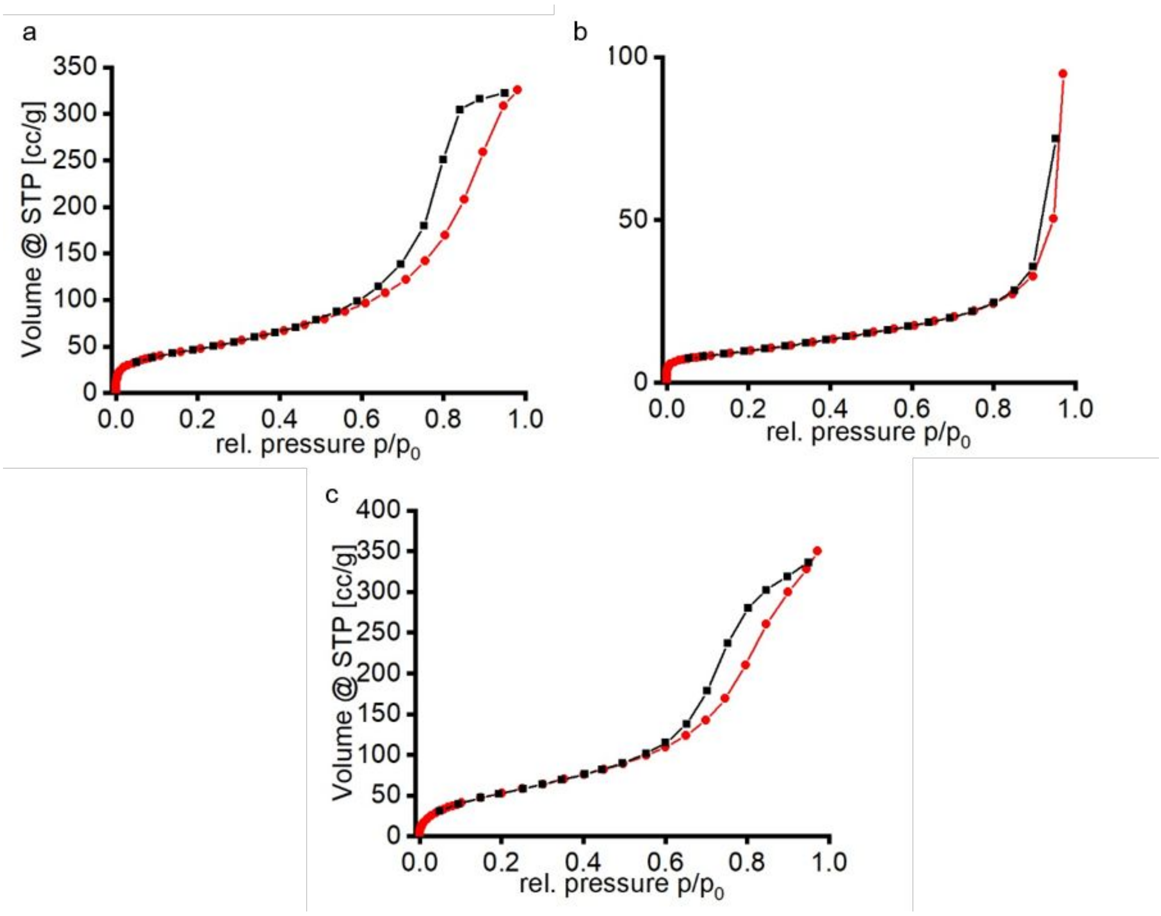

Figure S8. Ar Isotherms after calcination at 500 °C of a) L-titanate@Pd@L-titanate, b) Pd_{ext}@P25, and c) $Pd_{ext}@Al_2O_3$.

2.9 DRIFT spectra

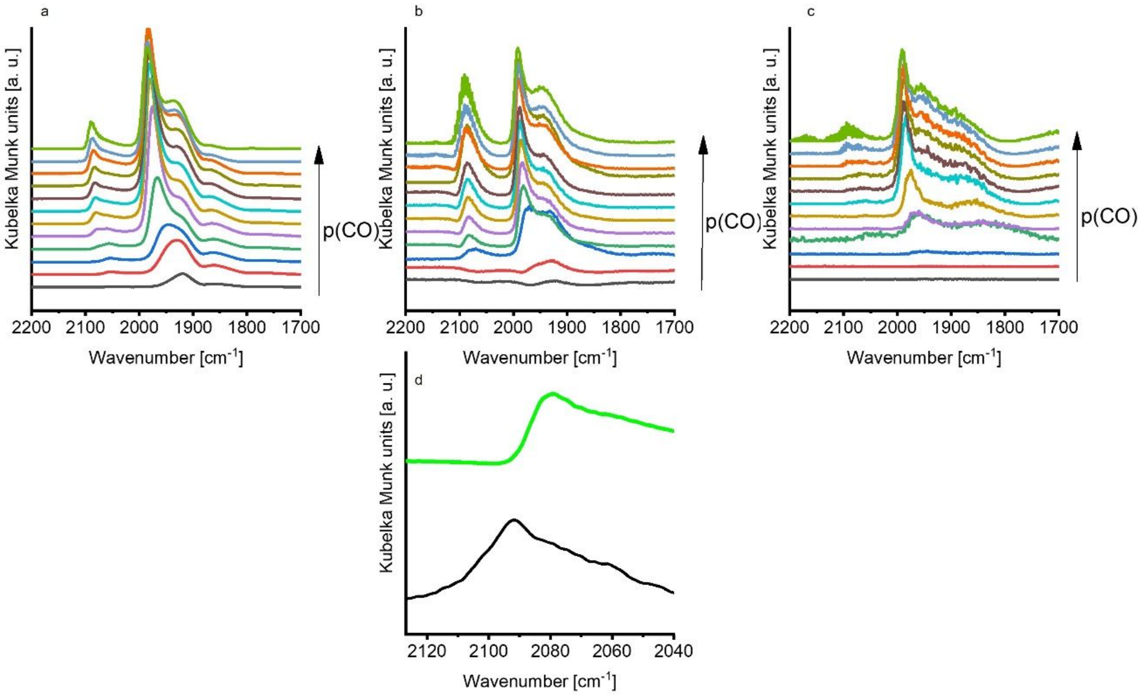

Figure S9. Drift spectra of a) Pd_{ext}@Al_2O_3, b) Pd_{ext}@P25, and c) L-titanate@Pd@L-titanate at increasing partial pressures of CO up to 60 mbar. d) zoom of the top region of L-titanate@Pd@L-titanate (black) and Pd_{ext}@Al_2O_3.

2.10 EEL spectra at the O K edge

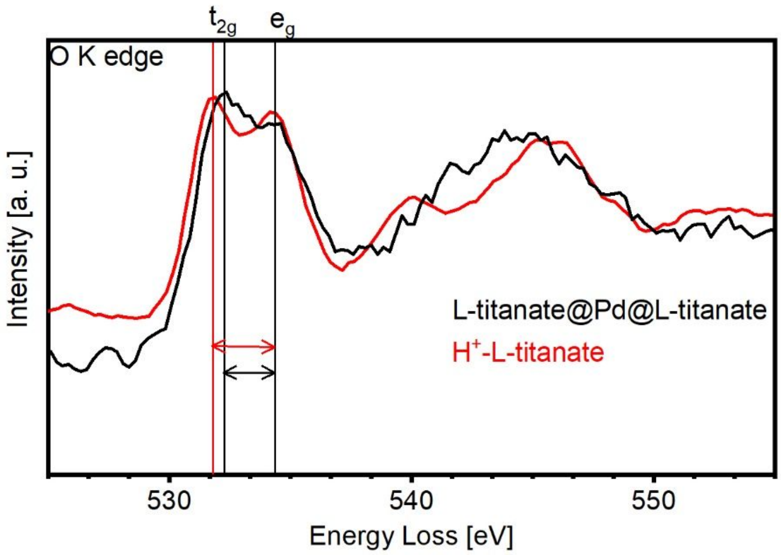

Figure S10. EEL spectra at the O K edge of L-titanate@Pd@L-titanate (black) and H^+-L-titanate (red) showing different extends of crystal field splitting.

2.11 Arrhenius plots

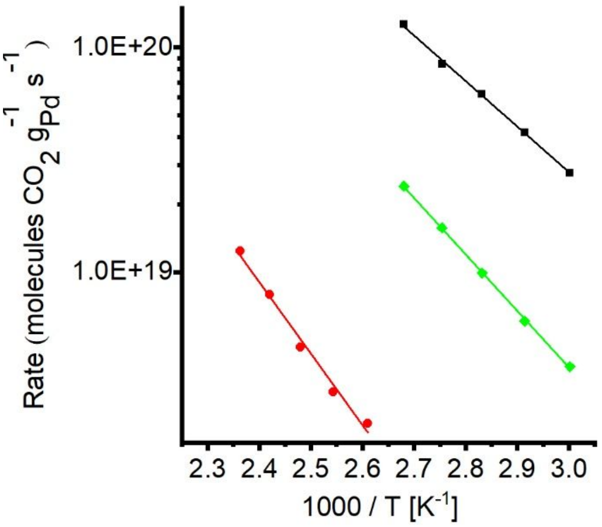

Figure S11. Arrhenius plots of L-titanate@Pd@L-titanate (black), Pd_{ext}@P25 (green), and Pd_{ext}@Al_2O_3 (red).

2.12 Evaluation of the kinetic experiments

The rate of mol of CO_2 produced per second was calculated as follows:

$$rate = \frac{Flow\ rate\ of\ CO \left[\frac{L}{min}\right] \cdot \frac{1\,mol}{22.4\,L} \cdot Conversion}{60 \left[\frac{s}{min}\right] \cdot g_{Pd}}$$

The change in rate was measured in terms of various partial pressures of CO and O_2. The kinetic equation is:

$$rate = k \cdot [CO]^a \cdot \ [O_2]^b$$

The partial pressure of one species was fixed while the other was changed in steps. The corresponding partial pressures of each step are listed in Table S2:

Table S 2. Gas partial pressures and reaction rates of L-titanate@Pd@L-titanate.

Point	ml/min (CO)	p(CO) [mbar]	ml/min (O_2)	p(O_2) [mbar]	Rate [mol CO_2 s^{-1} g^{-1}_{Pd}]
1	0.5	10.13	0.25	5.06	4.47607E-5
2	0.5	10.13	0.25	5.06	4.45398E-5
3	0.5	10.13	0.5	10.13	5.36554E-5
4	0.5	10.13	0.5	10.13	5.35515E-5
5	0.5	10.13	1.0	20.26	6.63204E-5
6	0.5	10.13	1.0	20.26	6.52704E-5
7	0.5	10.13	0.25	5.06	4.46675E-5
8	0.5	10.13	0.25	5.06	4.42985E-5
9	1.0	20.26	0.25	5.06	5.06409E-5
10	1.0	20.26	0.25	5.06	5.07298E-5
11	2.0	40.52	0.25	5.06	5.57622E-5
12	2.0	40.52	0.25	5.06	5.63976E-5

The calculation of *a* and *b* is demonstrated for the transition from point 2 to 3, when the partial pressure of O_2 is doubled.

$$\frac{4.45 \cdot 10^{-5} s^{-1} g^{-1}}{5.37 \cdot 10^{-5} s^{-1} g^{-1}} = \frac{k \cdot [10.13]^a \cdot \ [5.06]^b}{k \cdot [10.13]^a \cdot \ [10.13]^b} =$$

$$0.83 = 0.5^b$$

$$\ln(0.83) = \ln\left(0.5^b\right) = b \cdot \ln(0.5)$$

$$b = 0.27$$

The same calculations were done for the transition from point 4 to 5 which obtained *b* of 0.31. The average is 0.29.

The same calculations were done for variations of CO obtaining *a* of 0.13.

So the rate equation can be expressed as:

$$rate = k \cdot [CO]^{0.13} \cdot \ [O_2]^{0.29}$$

The calculations were done for $Pd_{ext}@Al_2O_3$ as well using the values from Table S 3:

Table S 3. Gas partial pressures and reaction rates of L-titanate@Pd@L-titanate

Point	ml/min (CO)	p(CO) [mbar]	ml/min (O_2)	p(O_2) [mbar]	Rate [mol CO_2 s^{-1} g^{-1}_{Pd}]
1	0.5	10.13	0.25	5.06	1.42724E-5
2	0.5	10.13	0.25	5.06	1.46914E-5
3	0.5	10.13	0.5	10.13	2.58875E-5
4	0.5	10.13	0.5	10.13	2.5091E-5
5	0.5	10.13	1.0	20.26	5.02798E-5
6	0.5	10.13	1.0	20.26	5.01251E-5
7	0.5	10.13	0.25	5.06	1.25558E-5
8	0.5	10.13	0.25	5.06	1.25683E-5
9	1.0	20.26	0.25	5.06	7.70412E-6
10	1.0	20.26	0.25	5.06	8.17334E-6
11	2.0	40.52	0.25	5.06	5.22527E-6
12	2.0	40.52	0.25	5.06	5.0282E-6

In this case the rate equation was determined to be:

$$rate = k \cdot [CO]^{-0.67} \cdot [O_2]^{0.91}$$

2.13. Long term stability of L-titanate@Pd@L-titanate

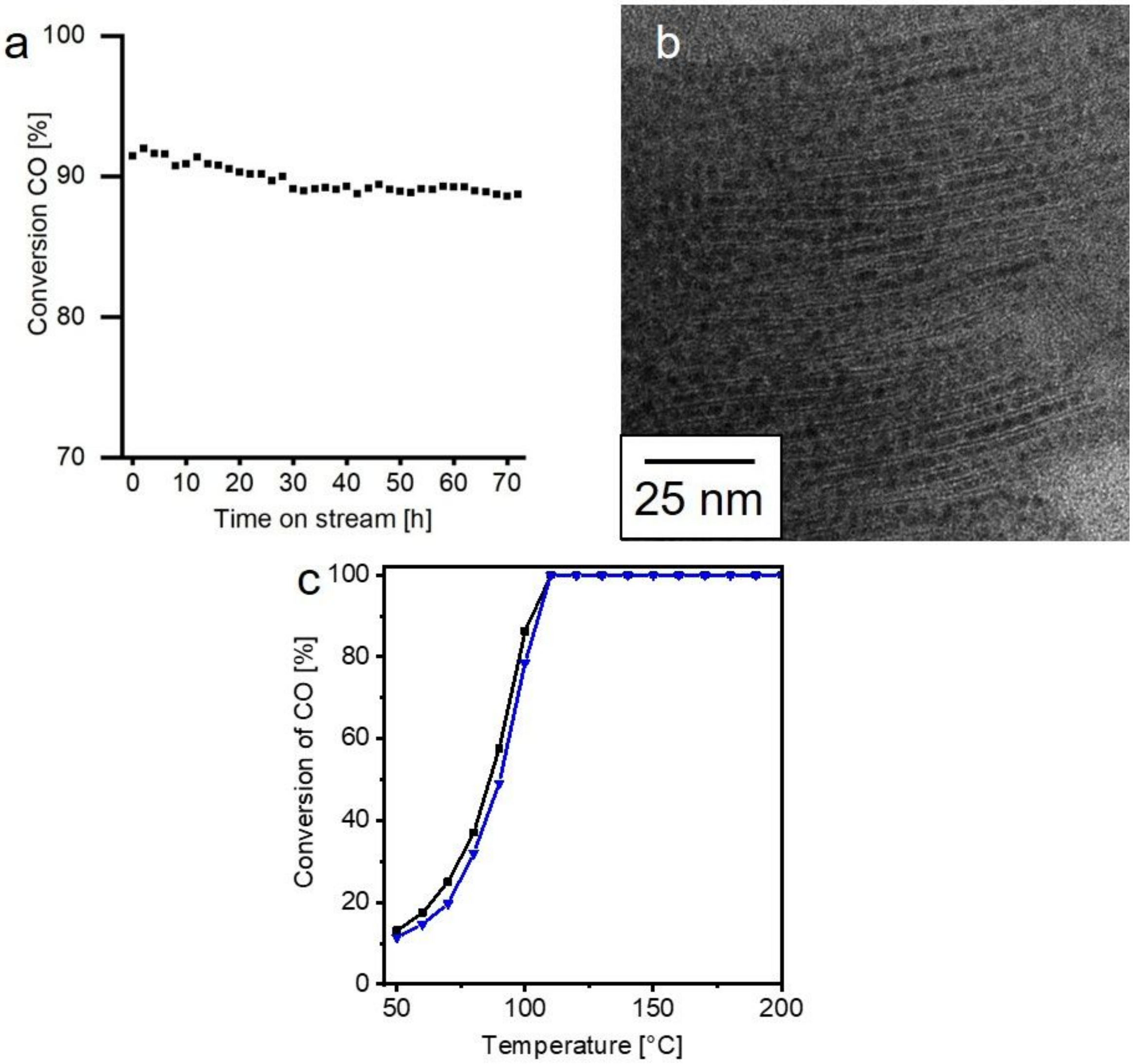

Figure S12. a) Stability test of L-titanate@Pd@L-titanate. Conditions: 100 °C, feed gas: 50 mL/min (1 vol% CO, 1 vol% O_2, balanced by N_2). b) TEM image of the used catalyst. C) Light-off curve of L-titanate@Pd@L-titanate after calcination at 500 °C for 5 h (black) and after calcination at 700 °C (blue).

3 References

(1) Flanagan, K. A.; Sullivan, J. A.; Müeller-Bunz, H. Preparation and Characterization of 4-Dimethylaminopyridine-Stabilized Palladium Nanoparticles. *Langmuir* **2007**, *23*, 12508-12520.

(2) Tanaka, T.; Ebina, Y.; Takada, K.; Kurashima, K.; Sasaki, T. Oversized Titania Nanosheet Crystallites Derived from Flux-Grown Layered Titanate Single Crystals. *Chem. Mater.* **2003**, *15*, 3564-3568.
